# Commentary: Interpreting diagnostic data on autism and gender dysphoria: clinical and research implications – a commentary on Sanders et al. (2025)

**DOI:** 10.1111/camh.70056

**Published:** 2025-12-16

**Authors:** Kai Thomas, Kate Cooper

**Affiliations:** ^1^ School of Psychology Cardiff University Cardiff UK; ^2^ Research Department of Clinical, Educational and Health Psychology University College London London UK; ^3^ Centre for Applied Autism Research, Department of Psychology University of Bath Bath UK

Sanders et al. (2025) make a valuable contribution to the literature by examining the co‐occurrence of autism and gender dysphoria diagnoses in a large, community‐based sample of children and adolescents. One of the most notable strengths of this study is its use of electronic health record data, encompassing over 750,000 individuals. Previous research has been limited to smaller samples within more specialist settings, such as academic medical centres. As well as exploring the prevalence of gender dysphoria among autistic and nonautistic participants, they investigated co‐occurring rates of depressive disorder, generalised anxiety disorder and suicide attempts. The study found that autistic children and adolescents were more likely to have recorded diagnoses of gender dysphoria than nonautistic children and adolescents. In addition, children and adolescents who were both autistic and had gender dysphoria were more likely to have higher rates of recorded diagnoses of depression, anxiety and suicide attempts compared to those who were not autistic and/or did not have a diagnosis of gender dysphoria.

The size of the sample allows for a robust comparison across four groups of young people (autistic only, gender dysphoria diagnosis only, autistic with a gender dysphoria diagnosis and nonautistic without a diagnosis of gender dysphoria), enabling insights into the prevalence and co‐occurrence of mental health difficulties associated with these intersecting identities. The findings underscore the clinical relevance of the co‐occurrence of autism and gender dysphoria. These results reinforce the importance of integrated, developmentally informed approaches to assessment and care.

While Sanders et al. (2025) provide valuable evidence of the association between autism and gender dysphoria in a large community sample, several limitations and further opportunities for inclusivity and nuance warrant attention. First, the reliance on formal diagnoses recorded in electronic health records needs to be carefully considered. We note that this is an inevitable consequence of using large health system data, which also contributes to the strength of the large, community sample. Many young people face barriers to accessing timely and accurate diagnoses of autism and gender dysphoria due to systemic inequities, lack of specialist services or stigma (Crane, Batty, Adeyinka, et al., 2018; Smith‐Young, Pike, Swab, & Chafe, 2025; Squires, Laws, Joubert, & Greenhill, 2024). It is not possible from the dataset to understand the clinical processes that led to diagnoses of autism, gender dysphoria, depression and anxiety. Diagnostic overshadowing may have occurred and diagnostic accuracy is likely to be dependent on clinician speciality (i.e. primary care, mental health and gender specialist). As such, the study may not accurately represent the prevalence of conditions reported.

The study focuses exclusively on the co‐occurrence of depression, anxiety and suicidality. This is a commendable aim, given the mental health needs of autistic youth and those with gender dysphoria. However, the inevitably small sample size in the autistic with gender dysphoria group (*n* = 59) means that these findings should be taken with caution. The analysis overlooked a wider range of mental health conditions that are known to co‐occur with both autism and gender dysphoria, such as eating disorders. This is particularly striking given the growing body of research highlighting elevated rates of disordered eating among autistic and gender‐diverse youth (Pham et al., 2021; Strauss et al., 2021). It is also important to consider the intersection of other neurodevelopmental diagnoses, such as attention deficit hyperactivity disorder (ADHD), which has also been found to co‐occur with both autism and gender dysphoria (Micai et al., 2023; Warrier, Greenberg, Weir, et al., 2020), and is linked to anxiety and depression in children and adolescents (Zhang, Liao, Rao, Gao, & Yang, 2025). Including such conditions would have offered a more comprehensive picture of mental health risk and service needs in this population.

It is notable that the prevalence of depression in the study by Sanders et al. (2025) was similar in nonautistic and autistic youth (at 2%), which is surprising given that most prevalence studies have identified that autistic youth are more likely to experience depression than nonautistic youth (Corbett, Muscatello, & McGonigle, 2024; Hollocks, Leno, Chandler, et al., 2023). It is possible that depression was being underdiagnosed or under recorded in this population, perhaps in cases where anxiety conditions were the primary diagnosis. However, Sanders et al. reported differences in rates of depression for youth with gender dysphoria. Specifically, those who were also autistic had a higher prevalence of depression compared to those with gender dysphoria who were not autistic. This could be due to a true difference, where the presence of co‐occurring autism and gender dysphoria increases the risk for mental health difficulties (and this would be supported by the adult literature, e.g. George & Stokes, 2018) and/or due to different diagnostic processes for youth experiencing gender‐related distress.

Sanders and colleagues identify that rates of suicide attempts were higher in those with gender dysphoria than in those without. Previous research has investigated rates of suicide in youth with gender dysphoria compared to those accessing mental health services and found similar rates of suicide attempts in these two groups (Ruuska, Tuisku, Holttinen, & Kaltiala, 2024). Clinicians must be aware that the risk of suicide attempts is elevated in youth with gender dysphoria compared to the general population and take appropriate steps to support them and their parents/carers. They should also be reassured that their risk management skills for supporting youth with mental health problems are likely to be highly transferable and supportive to youth experiencing gender dysphoria. Such risk management interventions should be individualised, considering the young person's social and environmental context, and include trusted adults in the young person's life to help keep them safe (Asarnow & Mehlum, 2019).

Given the dearth of research in this area, we note several opportunities for future research to build on the current findings. Firstly, future longitudinal studies are required to examine the impact of gender dysphoria on mental health across development. Examining these associations across time would also be important for interpreting potential cohort effects in the population, since awareness of gender dysphoria has increased over the last decade alongside an increase in diagnoses. Acknowledging this diagnostic gap is essential for interpreting these findings and considering their implications for service provision.

Future research should ensure that gender identity and sex assigned at birth are collected separately and accurately distinguished. Conflation of gender and sex risks misrepresenting the gender diversity of the sample and reinforces binary frameworks that may not reflect the lived experiences of trans, nonbinary and gender fluid children and young people. Longitudinal studies should also allow for gender identity to be collected at multiple points throughout the research to allow for the accurate representation of nonbinary and gender fluid identities, as well as acknowledging that gender identity can evolve and change for some young people across time. Accurately representing diverse gender identities, beyond binary trans identities, allows for outcomes to be disaggregated by gender identity. This is important given there is growing recognition of the mental health needs of nonbinary and gender‐expansive youth (Klinger et al., 2024).

Finally, future studies using large health system data should aim to provide comprehensive contextual information about the database and the population it serves. Information about socioeconomic status, insurance coverage or service features/availability would help readers assess the generalisability of the findings. Without this context, it is difficult to determine whether the observed patterns reflect broader trends or are shaped by specific features of the health system.

In conclusion, Sanders et al. (2025) provide a valuable contribution to the literature by examining the co‐occurrence of autism and gender dysphoria diagnoses in a large, community‐based sample of children and adolescents. Future studies should aim to expand these findings by examining these co‐occurring diagnoses across development to inform directional conclusions, including associations with additional mental health conditions and co‐occurring ADHD. Lastly, accurate measures of gender identity are important for ensuring evidence‐based clinical guidelines are nuanced and inclusive of all gender identities.

## Conflict of interest statement

The authors have no conflicts of interest to disclose.

## Funding information

K. T. is funded by a Health and Care Research Wales Advanced Fellowship award (2024–2027).

## Ethics statement

No ethics approval was required for this debate article.

## Data Availability

Data sharing is not applicable to this debate article as no data was created or analysed.

## References

[camh70056-bib-0001] Asarnow, J.R. , & Mehlum, L. (2019). Practitioner review: Treatment for suicidal and self‐harming adolescents–advances in suicide prevention care. Journal of Child Psychology and Psychiatry, 60, 1046–1054. 31512763 10.1111/jcpp.13130 PMC6880954

[camh70056-bib-0002] Corbett, B.A. , Muscatello, R.A. , & McGonigle, T. (2024). Trajectory of depressive symptoms over adolescence in autistic and neurotypical youth. Molecular Autism, 15, 18. 38698474 10.1186/s13229-024-00600-w PMC11064411

[camh70056-bib-0003] Crane, L. , Batty, R. , Adeyinka, H. , Goddard, L. , Henry, L.A. , & Hill, E.L. (2018). Autism diagnosis in the United Kingdom: Perspectives of autistic adults, parents and professionals. Journal of Autism and Developmental Disorders, 48, 3761–3772. 29948530 10.1007/s10803-018-3639-1 PMC6182717

[camh70056-bib-0004] George, R. , & Stokes, M.A. (2018). A quantitative analysis of mental health among sexual and gender minority groups in ASD. Journal of Autism and Developmental Disorders, 48, 2052–2063. 29362955 10.1007/s10803-018-3469-1

[camh70056-bib-0005] Hollocks, M.J. , Leno, V.C. , Chandler, S. , White, P. , Yorke, I. , Charman, T. , … & Simonoff, E. (2023). Psychiatric conditions in autistic adolescents: Longitudinal stability from childhood and associated risk factors. European Child & Adolescent Psychiatry, 32, 2197–2208. 35976471 10.1007/s00787-022-02065-9 PMC10576662

[camh70056-bib-0006] Klinger, D. , Oehlke, S.M. , Riedl, S. , White, P. , Yorke, I. , Charman, T. , … & Simonoff, E. (2024). Mental health of non‐binary youth: A systematic review and meta‐analysis. Child and Adolescent Psychiatry and Mental Health, 18, 126. 39385290 10.1186/s13034-024-00822-z PMC11465615

[camh70056-bib-0007] Micai, M. , Fatta, L.M. , Gila, L. , Caruso, A. , Salvitti, T. , Fulceri, F. , … & Scattoni, M.L. (2023). Prevalence of co‐occurring conditions in children and adults with autism spectrum disorder: A systematic review and meta‐analysis. Neuroscience & Biobehavioral Reviews, 155, 105436. 37913872 10.1016/j.neubiorev.2023.105436

[camh70056-bib-0008] Pham, A. , Kasenic, A. , Hayden, L. , Inwards‐Breland, D.J. , Sumerwell, C. , Twible, H. , … & Orlich, F. (2021). A case series on disordered eating among transgender youth with autism spectrum disorder. Journal of Adolescent Health, 68, 1215–1219. 10.1016/j.jadohealth.2020.12.143 33707147

[camh70056-bib-0009] Ruuska, S. , Tuisku, K. , Holttinen, T. , & Kaltiala, R. (2024). All‐cause and suicide mortalities among adolescents and young adults who contacted specialised gender identity services in Finland in 1996–2019: A register study. BMJ Mental Health, 27, e300940. 10.1136/bmjment-2023-300940 PMC10875569 38367979

[camh70056-bib-1010] Sanders, M. , Saade, Z. , & Mortillaro, G. (2025). Short Research Article: Autism spectrum disorder and gender dysphoria among adolescents in a large, integrated health system. Child and Adolescent Mental Health. 10.1111/camh.70042 41102019

[camh70056-bib-0010] Smith‐Young, J. , Pike, A. , Swab, M. , & Chafe, R. (2025). Parents' and guardians' experiences of barriers and facilitators in accessing autism spectrum disorder diagnostic services for their children: A qualitative systematic review. JBI Evidence Synthesis, 23, 6–68. 39474706 10.11124/JBIES-23-00332

[camh70056-bib-0011] Squires, L. , Laws, A. , Joubert, H.E. , & Greenhill, B. (2024). “Grasping on a spider web of hope”: Psychological challenges and impact of waiting to access UK gender dysphoria clinics on young adults. International Journal of Transgender Health, 26, 1255–1274. 41180918 10.1080/26895269.2024.2411525 PMC12573521

[camh70056-bib-0012] Strauss, P. , Cook, A. , Watson, V. , Winter, S. , Whitehouse, A. , Albrecht, N. , … & Lin, A. (2021). Mental health difficulties among trans and gender diverse young people with an autism spectrum disorder (ASD): Findings from trans pathways. Journal of Psychiatric Research, 137, 360–367. 33761424 10.1016/j.jpsychires.2021.03.005

[camh70056-bib-0013] Warrier, V. , Greenberg, D.M. , Weir, E. , Buckingham, C. , Smith, P. , Lai, M.C. , … & Baron‐Cohen, S. (2020). Elevated rates of autism, other neurodevelopmental and psychiatric diagnoses, and autistic traits in transgender and gender‐diverse individuals. Nature Communications, 11, 3959. 10.1038/s41467-020-17794-1 PMC7415151 32770077

[camh70056-bib-0014] Zhang, Y. , Liao, W. , Rao, Y. , Gao, W. , & Yang, R. (2025). Effects of ADHD and ADHD medications on depression and anxiety in children and adolescents: A systematic review and meta‐analysis. Journal of Psychiatric Research, 181, 623–639. 39740618 10.1016/j.jpsychires.2024.12.022

